# Succinate metabolism and its regulation of host-microbe interactions

**DOI:** 10.1080/19490976.2023.2190300

**Published:** 2023-03-22

**Authors:** Yi-han Wei, Xi Ma, Jiang-Chao Zhao, Xiu-Qi Wang, Chun-Qi Gao

**Affiliations:** a College of Animal Science, South China Agricultural University, Guangdong Laboratory for Lingnan Modern Agriculture, Guangdong Provincial Key Laboratory of Animal Nutrition Control, National Engineering Research Center for Breeding Swine Industry, Guangzhou, China; b State Key Laboratory of Animal Nutrition, College of Animal Science and Technology, China Agricultural University, Beijing, China; c Department of Animal Science, Division of Agriculture, University of Arkansas, Fayetteville, AR, USA

**Keywords:** Succinate, gut microbiota, immune cells, gut–immune tissue axis, inflammation

## Abstract

Succinate is a circulating metabolite, and the relationship between abnormal changes in the physiological concentration of succinate and inflammatory diseases caused by the overreaction of certain immune cells has become a research focus. Recent investigations have shown that succinate produced by the gut microbiota has the potential to regulate host homeostasis and treat diseases such as inflammation. Gut microbes are important for maintaining intestinal homeostasis. Microbial metabolites serve as nutrients in energy metabolism, and act as signal molecules that stimulate host cell and organ function and affect the structural balance between symbiotic gut microorganisms. This review focuses on succinate as a metabolite of both host cells and gut microbes and its involvement in regulating the gut – immune tissue axis by activating intestinal mucosal cells, including macrophages, dendritic cells, and intestinal epithelial cells. We also examined its role as the mediator of microbiota – host crosstalk and its potential function in regulating intestinal microbiota homeostasis. This review explores feasible ways to moderate succinate levels and provides new insights into succinate as a potential target for microbial therapeutics for humans.

## Introduction

There is a close symbiotic relationship between the gut microbiota and the host. The gut microbiota helps maintain healthy host immune function by directly regulating intestinal mucosal immune cells, such as epithelial or dendritic cells (DCs), and by producing important immune metabolites. At the onset of an intestinal disorder, gut dysfunction occurs, which can lead to systemic diseases, such as diabetes,^1^ colitis,^2^ and rheumatic disorders.^3^ Hence, the crucial role of gut microbes in maintaining immune homeostasis has led to the emergence of new microbial therapeutics, such as fecal microbiota transplantation (FMT) and dietary intervention, to help maintain healthy gut homeostasis and reduce the risk of intestinal disorders.

Succinate is a circulating metabolite that helps regulate cellular nutrient metabolism and thus has potential application value in medical care. For instance, it promotes the deposition of skeletal muscle protein^4^ and regulates muscle fiber remodeling in the exercise state.^5,6^ During glucolipid metabolism, succinate regulates glucose homeostasis to ameliorate hyperglycemia in obese mouse models.^7^ It reduces white adipose tissue deposition in obese mouse models, thus exhibiting the potential to help prevent obesity.^8^ Furthermore, succinate has potential as a target for immune monitoring. Abnormal accumulation of succinate has been found in patients experiencing certain diseases, such as chronic inflammation,^9,10^ ischemia,^11^ and even cancer.^12,13^ Although it is unclear whether abnormal accumulation of succinate is a cause of these diseases, excessive accumulation of succinate can potentially increase the risk of disease progression.^14^

Succinate is an intermediate metabolite or end-product of many intestinal microorganisms. In industrial production, succinate can be extracted at a high yield from some modified microbes (e.g., *Actinobacillus succinogenes*,^15^ *Mannheimia succiniciproducens*,^16^ *Saccharomyces cerevisiae*,^17^ *Corynebacterium glutamicum*.^18^ In addition to its industrial application value, succinate is of great value in intestinal microbial therapeutics. It mediates the function of intestinal microorganisms by stimulating host mucosal immune cells and helps maintain a healthy balance between the gut microbiota. Herein, we summarize the role of succinate in modulating immune cell function, with particular attention to its role as a mediator of signal crosstalk between microbial metabolism and intestinal mucosal immune cell development. In addition to evaluating the potential of succinate to improve gut microbiota structure, we seek to understand the mechanism by which succinate affects intestinal homeostasis and to explore the potential of succinate as a microbiological therapy for the prevention and treatment of inflammation.

## Succinate synthesis and degradation in the gut

### Gut microbiota-produced succinate and its degradation pathway

**Figure 1.**
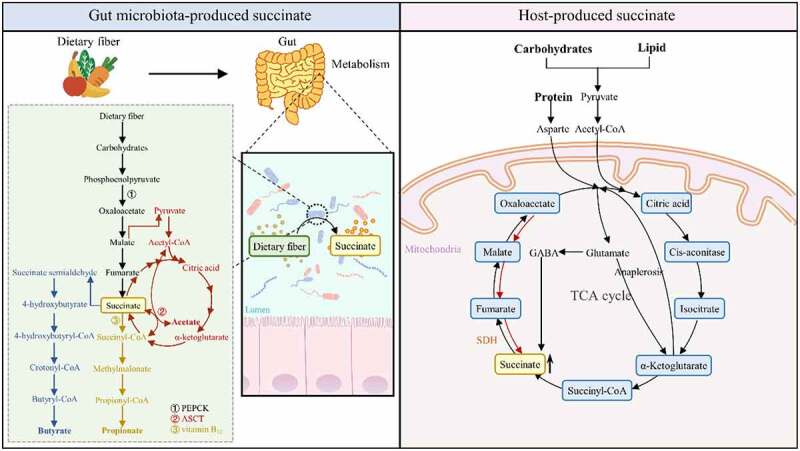
Synthesis and degradation of succinate by host and gut microbiota. (1) the left part shows the synthesis and degradation of gut microbiota-produced succinate. During this process, the gut microbiota metabolizes dietary fiber into succinate. As presented in the green part, succinate is the intermediate product of gut microbiota-produced SCFAs. The black line represents the biosynthetic pathway of succinate in the gut microbiota. The blue line represents the pathway through which succinate is metabolized to butyrate. The yellow line represents the degradation pathway of succinate to propionate. The red line represents the conversion relationship between succinate and acetate. (2) the right part shows the synthesis and degradation of host-produced succinate. Within a series of biochemical steps, carbohydrates, lipids, and proteins ultimately participate in the mitochondrial TCA cycle to generate energy. Succinate is generated during this process.

Succinate is the intermediate metabolite in the fermentation of indigestible dietary and host-derived carbohydrates into short-chain fatty acids (SCFAs) by gut microbes and provides critical energy substrates for cell proliferation, such as with intestinal epithelial cells (IECs). *Bacteroides spp*., *Prevotella spp*., *Firmicutes spp.*, and other bacteria in the intestinal tract can metabolize most pentose and hexose carbohydrates to produce succinate (Figure 1).^19,20^

### Gut microbiota succinate – propionate pathway

Most intestinal microorganisms, such as *Bacteroides spp*., *Prevotella spp*., *Firmicutes spp.*, and *Veillonella spp.*, metabolize indigestible dietary carbohydrates into propionate through the succinate pathway. During this process, carbohydrates are converted into phosphoenolpyruvate (PEP), which is then converted to oxaloacetate (OAA) by phosphoenolpyruvate carboxykinase (PEPCK) in the presence of carbon dioxide (CO_2_). With the activation of malate dehydrogenase and fumarate dehydrogenase, OAA is then converted to succinate. Subsequently, succinate is converted to succinyl-CoA and, with the participation of vitamin B_12_, to methylmalonate (MMA), which is further converted to propionate.^20,21^ Although succinate is an intermediate metabolite produced in low quantities during this process, it can accumulate under the influence of PEPCK and vitamin B_12_.^22^ PEPCK is a key rate-limiting enzyme in this pathway and is affected by CO_2_ levels.^23^ Under high CO_2_ (CO_2_: glucose = 1:1 mol), PEPCK can fix CO_2_ to synthesize oxaloacetic acid, while at low CO_2_ (CO_2_: glucose = 1:10 mol), PEP is mainly converted to pyruvate via pyruvate kinase and eventually produces lactate and formate. Therefore, increasing the concentration of CO_2_ can increase the activity of PEPCK to promote succinate production. Vitamin B_12_ is a cofactor of methylmalonyl-CoA mutase (MCM), which catalyzes the reversible isomerization of succinyl-CoA to MMA. Limiting vitamin B_12_ levels can control the production of succinate. According to experimental evidence, an accumulation of succinate was found in vitamin B_12_-depleted rumen *Prevotellaceae* cultures.^24^

### Gut microbiota succinate – acetate pathway

Some bacterial species, such as *Propionibacterium granulosum*, not only generate propionate via the succinate pathway but also produce acetate by utilizing succinate, as a byproduct of the succinate pathway. This process is achieved by acetate:succinate CoA-transferase (ASCT).^25^ Mechanistically, these bacteria feed on food in the gut and synthesize and store glycogen during host feeding. During host fasting, the bacteria metabolize glucose into malate. Malate is either converted to propionate by the succinate pathway or converted to pyruvate, which is then converted to acetyl-CoA. ASCT transfers the CoA portion of acetyl-CoA to succinate, producing succinyl-CoA and acetate, and can also generate succinate by utilizing acetate as a substrate. The bacteria *Acetobacter aceti* can use ASCT to complete the TCA cycle; that is, ASCT replaces the canonical TCA cycle succinyl-CoA synthetase (SCS) to convert succinyl-CoA into succinate to complete the TCA cycle.^26^ This can provide a new way to increase the yield of succinate using acetate as a raw material in industry.

### Gut microbiota succinate – butyrate pathway

Some gut microorganisms can also convert succinate to butyrate, such as *Prevotellaceae*, and their conversion from carbohydrate to succinate is similar to that of propionate-producing bacteria through the succinate pathway described previously. The difference in butyrate producers is that succinate is converted to succinyl-CoA, which is then changed into succinate semialdehyde by succinate semialdehyde dehydrogenase. Subsequently, butyrate is synthesized through a series of reactions involving 4-hydroxybutyrate, 4-hydroxybutyryl-CoA, crotonyl-CoA, and butyryl-CoA.^22^

### Host-produced succinate and its degradation pathway

Succinate is produced and metabolized by the tricarboxylic acid cycle (TCA) in the mitochondria of host cells during the metabolic production of carbohydrates, proteins, and fats. As an intermediate metabolite of the TCA cycle, succinate is generated from α-ketoglutarate (AKG) by 2-oxoglutarate dehydrogenase (OGDH) and SCS. Additionally, succinate can be synthesized from glutamine and used through anaplerosis to produce AKG.^27^ Moreover, succinate can be produced without passing through the TCA cycle. For example, glutamate is converted to succinic semialdehyde by the “γ-aminobutyric acid (GABA) shunt”, which is then converted by succinic semialdehyde dehydrogenase and vitamin B_12_ to succinate,^28^ after which succinate is oxidized to fumarate by succinate dehydrogenase (SDH). These pathways help to maintain succinate at an appropriate circulation level (Figure 1).

## The interaction between succinate and gut microbiota

### Dietary succinate promotes some gut microbiota colonization

**Figure 2.**
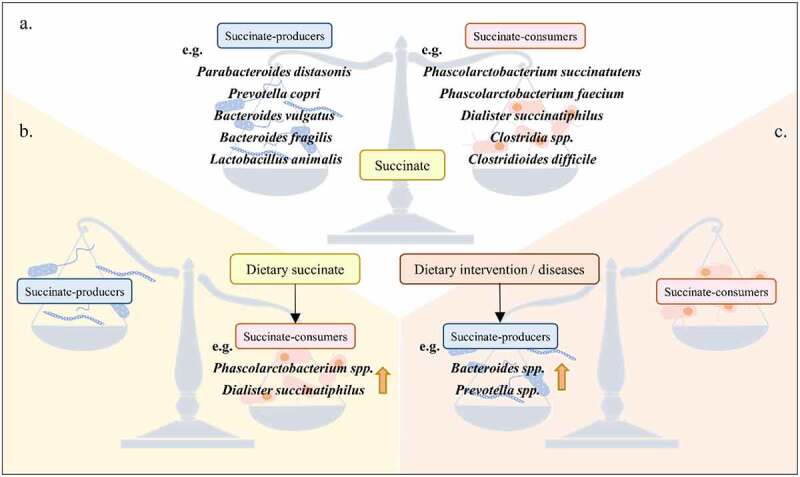
The interaction between succinate and gut microbiota. (A) in the healthy state, the number of succinate-producing bacteria and succinate-consuming bacteria related to succinate levels is in a dynamic equilibrium. (B) Dietary succinate can tip the balance and increase the number of bacteria that utilize succinate. (C) Dietary intervention with high protein, fat and fiber can increase the number of succinate-producing bacteria, and it is often accompanied by an increase in the number of succinate-producing bacteria under the pathological state of the host.

In addition to being a precursor of SCFAs, succinate can also be used by certain succinate-consuming bacteria as a nutrient for proliferation. Some gut microbiota, instead of using carbohydrates for metabolism, utilize succinate as a substrate to obtain a constant source of energy (Figure 2). *Phascolarctobacterium succinatutens* consumes succinate to produce propionate.^29^ This may be the same as *Phascolarctobacterium faecium*, which lacks fumarate reductase required for succinate production, so it cannot produce succinate and needs to utilize succinate produced by other bacteria to complete its own metabolism.^30^ In addition, *Clostridia spp*. colonization can be promoted by treating drinking water with succinate in mice. This is probably because succinate can consume oxygen to promote the colonization of strict anaerobes.^31^ However, some gut microbiota that are detrimental to host health and some pathogens can also utilize succinate to facilitate their own survival (Figure 2). *Clostridioides difficile*, a bacterium that causes infection of the colon, utilizes succinate produced by other bacteria (e.g., *Bacteroides spp*.) to proliferate. In this process, succinate does not directly produce ATP to provide energy but instead acts as an electron sink to enable the oxidation of the electron carrier. Consequently, the conversion of succinate to butyrate enables NADH regeneration to NAD+, which is required for the catabolism of sugar alcohols, sorbitol, and other dietary sugars.^32^ *Salmonella Typhimurium* can take up and utilize gut microbiota-produced succinate to complete its TCA cycle, thus competing with the microbiota and colonizing the intestine.^33^ Based on the above studies, we summarize that dietary succinate can shape gut microbiota composition, that is, increase the proportion of succinate-consuming bacteria. Although some studies have reported that dietary succinate can indeed increase the proportion of succinate consumers (e.g., *Phascolarctobacterium spp*. and *Dialister succinatiphilus*),^34,35^ it is worth pondering that there are many kinds of succinate-consuming bacteria, and different bacteria use succinate to complete their own metabolism for different purposes. Therefore, the commonalities and differences of colonies using succinate metabolism need to be further studied.

### Changes in gut microbiota composition alter succinate levels

Dietary intervention can adjust the composition of the gut microbiota, thereby affecting host health through gut microbiota-produced metabolites (Figure 2).^36^ High intake of fat and protein promotes the colonization of *Bacteroides spp*., while high intake of fiber promotes *Prevotella spp*. proliferation.^37^ These bacteria can produce succinate. It has been found that the tilt of dietary structure toward a certain nutrient (i.e., a high-fat, high-protein and high-fiber diet) increases succinate-producing bacterial colonization and thus succinate production.^38–40^ In the case of specifically altered gut microbiota composition, further dietary intervention promotes the production of metabolites by specific colonizing bacteria. For example, after increasing the colonization of succinate-producing bacteria such as *Parabacteroides distasonis*^7^ and *Prevotella copri*,^41^ a high-fiber diet can enhance succinate levels. This suggests a way in which dietary interventions can be combined with microbiota transplantation to specifically modulate the gut microbiota structure, thereby adjusting succinate levels.

Many diseases are closely related to gut dysbiosis. Gut dysbiosis interrupts the balance of symbiotic microbiota and thus adversely affects host health (Figure 2). In recent years, studies have reported that the increasing proportion of succinate-producing bacteria becomes a detriment to the host’s health; that is, the relative abundance of succinate-producing microbiota members (e.g., *Bacteroidaceae, Prevotellaceae* and *Veillonellaceae*) is higher than that of succinate-consuming microbiota members (e.g., *Phascolarctobacterium spp., Odoribacteraceae* and *Clostridaceae*), resulting in abnormally increased succinate in inflammatory bowel disease (IBD), Crohn’s disease (CD) and ischemia/reperfusion (I/R) patients and mouse models.^42–44^ In addition to gut-related diseases, a similar scenario has been observed in human obesity,^45^ imiquimod-induced psoriasis mice^46^ and weanling piglets with diarrhea.^47^ Based on the above studies, it can be inferred that under metabolic abnormalities and disease circumstances, gut microbiota composition is biased toward an increase in the proportion of succinate-producing bacteria, which will lead to more succinate production. This further emphasizes the key role of succinate in affecting host health and immune function.

## The mechanism of succinate in regulating the gut – immune axis

**Figure 3.**
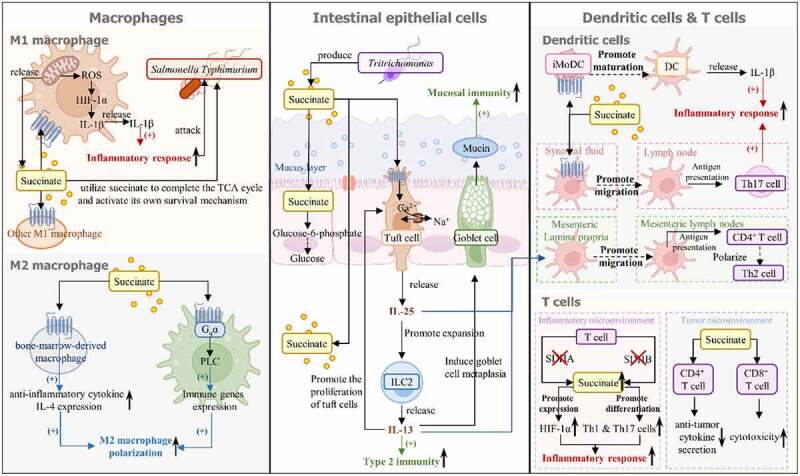
Succinate regulates the function of mucosal immune cells in the intestine. Succinate affects the functions of intestinal macrophages, tuft cells, and dendritic cells (DCs): (1) the left section shows that succinate regulates the function of both M1 and M2 macrophages. Specifically, increased concentrations of succinate in M1 macrophages promote HIF-1α production. This further promotes macrophage release IL-1β, a proinflammatory cytokine, thus triggering the inflammatory response. Succinate produced by M1 macrophages can also bind to SUCNR1 on neighboring M1 macrophages to regulate the same inflammatory response. The inflammatory response of macrophages can attack *Salmonella Typhimurium*, but *Salmonella Typhimurium* can also utilize succinate secreted by macrophages. In addition, succinate has the potential ability to promote M2 macrophage polarization. By binding to SUCNR1 expressed in BMDMs, succinate can induce IL-4, which promotes M2 phenotype differentiation. Succinate also stimulates M2 phenotype polarization via SUCNR1-activated G_q_ signaling in M2 macrophages. (2) the middle section shows that gut microbiota-produced succinate can cross IECs via the SLC13A family expressed on epithelial cells into the lamina propria and can be metabolized into glucose in IECs. Furthermore, *Tritrichomonas*-generated succinate binds to SUCNR1 on tuft cells and stimulates them to release IL-25, which acts on ILC2s to promote the secretion of IL-13. IL-13 directly enhances type 2 immunity, acts on DCs and promotes their migration into the mesenteric lymph nodes. This induces the polarization of CD4+ T cells into Th2 cells, thereby indirectly enhancing type 2 immunity. In addition, IL-13 promotes tuft cell proliferation and activates goblet cell transformation to increase the amount of mucin, thus enhancing mucosal immunity. (3) the right section shows that succinate regulates the antigen presentation and inflammatory function of DCs. Specifically, succinate acts on iModcs expressing relatively high levels of SUCNR1 and can promote the maturation of iModcs and the migration of mature DCs into the lymph nodes. Exogenous succinate can also enhance antigen presentation by DCs. Moreover, succinate generated by mature DCs has the same function as exogenous succinate in promoting the release of IL-1β from macrophages. Furthermore, succinate influences T-cell function. In the inflammatory microenvironment, SDHA or SDHB deficiency causes increases in succinate level and changes in T-cell metabolism, thus promoting the inflammatory response. In the tumor microenvironment, succinate inhibits CD4+ T cells from secreting antitumor cytokines but enhances the cytotoxicity of CD8+ T cells.

The commensal gut microbiota has a mutualistic relationship with the host. Gut microbes can sense the state of the host’s gut immune system. They can directly promote and regulate intestinal mucosal immunity and indirectly activate the immune defense function through metabolites to target pathogens. Changing succinate concentrations are closely linked to host health. Deviation of the succinate metabolites of the host from normal levels can affect the composition of the microbiota, leading to dysbiosis during immune disorders. Under normal physiological conditions, succinate concentrations in the intestinal lumen and feces are between 1–3 μM (or μmol/g).^33^ Although the specific concentrations may vary by host species and sample type, these succinate levels are considered low. However, an abnormally increased concentration of to 7–25 mM succinate was found in the feces of patients with IBD.^48^ Moreover, succinate is maintained at 5 μM in healthy plasma^49^ but has been found to accumulate abnormally up to 5–9 mM in pathological conditions (e.g., inflammation and cancer).^9,50^ Hence, succinate may play a key role in the interaction between the gut microbiota and host intestinal immunity. Mechanistically, succinate mainly regulates host immune function by regulating immune cells such as macrophages, DCs and IECs. Herein, we focus on the effects of succinate on these three immune cells and the mechanisms thereof to provide a reference for further research on the gut microbiota – succinate–host immune regulation axis (Figure 3).

### Succinate and macrophages

Classically activated M1 macrophages secrete proinflammatory cytokines (e.g., IL-6, IL-1β, and interferons-γ) to trigger a type 1 response, which involves antimicrobial effectors that regulate the activation of phagocytes^51^ and acute inflammation^52^ to inhibit the pathogen’s spread. Increased succinate is related to the polarization of macrophages toward the M1 phenotype. Succinate can participate in and facilitate the proinflammatory process of M1 macrophages and can be used as a marker of M1 proinflammatory activity. Specifically, M1 macrophages undergo metabolic reprogramming, represented by an increase in glycolysis, which inhibits the activity of isocitrate dehydrogenase (IDH) and succinate dehydrogenase (SDH) in the TCA cycle, causing TCA cycle fragmentation with citric acid and succinate accumulation. SDH links the succinate/fumarate couple to the coenzyme Q (CoQ) pool, and the oxidation of succinate to fumarate by SDH is coupled with the reduction of ubiquinone (UQ) to ubiquinol (UQH_2_). The close midpoint potential of the UQ/UQH_2_ and fumarate/succinate couples causes electrons to flow in any direction between the Krebs cycle and the CoQ pool during SDH catalysis. When the mitochondrial respiratory chain oxidizes the accumulated succinate in M1 macrophages under a high proton motive force, the CoQ pool decreases, resulting in the production of electrons that are driven backward into complex I instead of entering complex III. This reverse electron transport (RET) drives large amounts of reactive oxygen species (ROS) production. ROS can mediate the oxidation of Fe^2+^ into Fe^3+^, inhibiting the activity of proline hydroxylase (PHD), which is dependent on Fe^2+^,^53^ further activating hypoxia-inducible factor-1α (HIF-1α) to promote IL-1β secretion.^27^ At the same time, succinate released into the extracellular milieu binds with succinate receptor 1 (SUCNR1) on either the same or neighboring macrophages to enhance the release of IL-1β in the inflammatory microenvironment.^54^ Thus, diverse sources of succinate jointly promote the proinflammatory effect of M1 macrophages. However, enteric pathogens, such as *Salmonella Typhimurium*, have evolved to detect increased succinate during the metabolic reprogramming process of M1 macrophages and utilize the succinate generated during the proinflammatory response to complete the TCA cycle and activate their own survival mechanism.^55^ Furthermore, M1 macrophages in an inflammatory microenvironment overreaction aggravate inflammatory diseases, such as IBD^56,57^ and rheumatoid arthritis (RA),^58^ and are accompanied by an abnormal increase in succinate concentration. These results reveal that the effect of succinate on the proinflammatory function of M1 macrophages is a double-edged sword: succinate promotes acute inflammation to defend against pathogens, but it may also promote the growth of some pathogens and worsen inflammatory diseases, thus endangering host health.

In contrast, alternatively activated M2 macrophages secrete immunosuppressive cytokines (e.g., IL-10 and TGF-β) to promote a type 2 immune response, which mobilizes gut immune cells to eliminate invasive pathogens and stimulates an anti-inflammatory response.^51,59^ After acute inflammation clears the infection, the body will undergo the resolution of inflammation to repair the damaged tissue and return to homeostasis. In the resolution stage, macrophages shift from M1 to M2 macrophages to exert anti-inflammatory functions.^60^ This property leads macrophages to be considered a prospective novel therapeutic approach to control IBD in light of findings that some current IBD therapies are associated with the increasing accumulation of M2 macrophages.^60^ Although Mills et al. showed that succinate decreased the expression of M2 macrophage-related genes,^61^ recent studies have also found that succinate is produced as part of the inflammatory program and promotes the anti-inflammatory response of macrophages through SUCNR1 as a negative feedback signal. Research has shown that succinate-treated macrophages and peritoneal mast cells can alleviate colitis,^62^ and SUCNR1 is highly upregulated in M2-phenotype differentiation but not when shifted toward M1 macrophages.^63^ In the adipose tissue of a healthy body, succinate was found to enhance the expression of IL-4, an anti-inflammatory cytokine, via SUCNR1 signaling to bone marrow-derived macrophages (BMDMs), promoting polarization to M2 macrophages.^64^ Moreover, extracellular succinate enhances the transcription of immune function genes that are preferentially expressed in M2 macrophages to facilitate M2 phenotype polarization via SUCNR1-phospholipase C (PLC)-inositol trisphosphate (IP3)-Ca^2+^ signaling.^65^ These findings suggest that the succinate – SUCNR1 axis is indispensable for the anti-inflammatory response of macrophages. Whether the succinate-SUCNR1 signaling pathway acts as an anti-inflammatory mediator or promotes inflammation may depend on the inflammatory status of SUCNR1-expressing cells. Although there are few reports on the specific mechanism by which succinate exerts anti-inflammatory effects in intestinal M2 macrophages, the aforementioned findings shed light on the new possibility of the application of succinate in controlling inflammatory disease by promoting M2-phenotype polarization. Remarkably, research has also reported that an increased ratio of AKG/succinate promotes the activation of M2 macrophages, while a lower ratio boosts the activation of M1 macrophages.^66^ This suggests that although succinate has the potential to promote the polarization of M2 macrophages, its dynamic level changes are closely related to the dynamic changes in other metabolites. The specific succinate concentration that can determine the polarization direction of macrophages needs to be further studied.

### Succinate and dendritic cells and T cells

DCs are a class of specialized antigen-presenting cells (APCs) that act as a bridge between innate and adaptive immunity, helping to maintain homeostasis. Immature DCs take up antigens and recognize pathogen-associated molecular patterns of invading microorganisms or products of injured or dead host cells through Toll-like receptors (TLRs) and other microbial sensors.^67^ This process triggers DCs to mature, migrate into the T-cell zone of lymph nodes, and bind to and activate T cells. The mature DCs also release proinflammatory cytokines.

SUCNR1 was found to be highly expressed in immature monocyte-derived DCs (iMoDCs), but its expression decreased as DCs matured.^68^ Thus, succinate was subsequently found to have a direct impact on the immune function of iMoDCs, and these functions are dependent on SUCNR1 signaling. Mechanistically, succinate binds to SUCNR1 on iMoDCs to induce intracellular calcium mobilization and extracellular signal-regulated kinases 1 and 2 (ERK1/2) phosphorylation and synergizes with TLRs to promote iMoDC maturation and IL-1β release to enhance the inflammatory response to defend against invading pathogens. Additionally, succinate enhances the migratory ability of DCs and antigen-specific activation of T cells, but these promotive effects are attenuated when SUCNR1 is knocked out.^68^ In line with the inflammatory effect of succinate on macrophages, succinate has opposite effects on the body in states of health or inflammation. For example, accumulated extracellular succinate in the synovial fluid (SF) of RA patients^69,70^ binds with SUCNR1 to trigger the process that guides DC migration into the lymph nodes. This results in an increased frequency of T helper 17 (Th17) cells, which are related to articular lesions and can exacerbate inflammation, bone erosion, the mechanical hyperalgesia response, and neutrophil infiltration in the joint.^71^

Of note, HIF-1α has been found to play a key role in how DCs influence inflammatory diseases. In a colitis mouse model, a lack of HIF-1α in gut DCs exacerbates colitis.^72^ In obesity mouse models, HIF-1α deficiency in adipose tissue DCs was found to increase adipose tissue inflammation and atherosclerotic plaque formation.^73^ These studies suggest that selectively activating HIF-1α in DCs could help ease the overreaction of the inflammatory response. It has been shown that succinate can induce HIF-1α expression through a SUCNR1-dependent pathway;^74^ thus, succinate may hold promise as a new strategy for targeting DCs to treat inflammation. For example, using succinate to target intestinal DCs may be an attractive therapeutic option for IBD.

Succinate not only indirectly affects T-cell function by affecting DCs but also directly regulates T-cell function in the inflammatory microenvironment. SDH gene expression and enzyme activity increase significantly during T-cell activation, and SDH deficiency can inhibit the survival and proliferation of T cells. In T-cell-mediated mouse and human colitis models, SDHA deficiency in IECs leads to changes in T-cell metabolism, disrupting the oxidative phosphorylation (OXPHOS) of IEC cells and leading to increased levels of succinate, which may further promote HIF-1α expression and thus promote inflammation.^75^ Similarly, SDHB-deficient T cells exhibit increased succinate/AKG ratios, induce proinflammatory gene markers, and promote the differentiation of Th1 and Th17 lineages, which are proinflammatory T cells.^76^ In the tumor microenvironment, loss of function of SDH leads to an abnormal increase in succinate levels. Succinate is taken up by CD4+ T cells through MCT1, thus inhibiting SCS activity and impairing glucose flux. This results in the inhibition of antitumor cytokine secretion.^77^ Succinate can also bind to SUCNR1 on CD8+ T cells to promote their killing function, but when protein C activity decreases due to lactate accumulation, it will block succinate-SUCNR1 signaling to reduce the cytotoxicity of CD8+ T cells.^78^ Although there are few studies on the relationship between succinate and T cells, these studies suggest that succinate plays an important role in the adaptive immune system. Further studies on the role of succinate in T-cell-related diseases are expected to provide new strategies for the treatment of these diseases.

### Succinate and intestinal epithelial cells

IECs, including absorptive enterocytes and secretory cells (i.e., tuft cells, goblet cells, Paneth cells, and enteroendocrine cells),^79,80^ are a key part of the host immune barrier and are considered immune cells.^81^ IECs are the boundary between the lumen and lamina propria and serve as a bridge between the components of these two microenvironments. Gut microbiota-produced succinate plays a key role in this communicational process.

Succinate helps regulate the host metabolism as a precursor of intestinal gluconeogenesis, thus aiding glucose homeostasis in IECs. Succinate secreted by bacteria (i.e., *Parabacteroides distasonis* and *Prevotellaceae*) acts as a substrate for fructose-1,6-bisphosphatase (FBPase), a rate-limiting enzyme involved in intestinal gluconeogenesis. This step regulates the conversion of glucose into endogenous glucose, which improves the homeostasis of host glucose.^7,40^ Furthermore, gut microbiota-produced succinate can cross IECs via the SLC13A family expressed on epithelial cells into the lamina propria to directly activate M1 macrophages and other immune cells.^42^ Additionally, succinate secreted by *Tritrichomonas* has been found to promote tuft cell proliferation and induce type 2 immunity.^82,83^ It has been confirmed that the numbers of tuft cells in the inflammatory ileal tissues of CD patients and mice were significantly lower than those in the corresponding tissues of healthy controls,^84^ suggesting that increasing the population of tuft cells benefits intestinal health. Thus, succinate, as a metabolite of gut microbiota, performs crucial roles in stimulating host immunity. The underlying mechanism is that *Tritrichomonas*-generated succinate binds to SUCNR1, which is expressed on tuft cells,^85^ leading to the activation of intracellular Ca^2+^ flux to open transient receptor potential cation channel subfamily M member 5 (TRPM5), which then causes Na+ influx and membrane potential depolarization.^82,83^ Subsequently, tuft cells act on type 2 innate lymphoid cells (ILC2s) by releasing IL-25 to promote ILC2 expansion and to stimulate ILC2s to release IL-13. IL-13 promotes tuft cell proliferation to form a feed-forward loop^86–88^ and induces goblet cell metaplasia, which results in the secretion of mucins^89^ and certain factors that facilitate intestinal defense, such as resistin-like protein β (RELMβ).^90,91^ Together, these factors enhance the defense against pathogen invasion mediated by the mucus layer. The succinate – tuft cell axis also indirectly influences mucosal immune cells in the lamina propria, such as DCs, by secreting IL-25 and IL-13. IL-25 produced by tuft cells acts on DCs to regulate the polarization of T cells into Th2 cells.^92^ IL-13 produced by ILC2s activates DCs in the mesenteric lamina propria to migrate into the mesenteric lymph nodes, polarizing naive CD4+ T cells into Th2 cells and thus promoting type 2 immunity.^93^

Notably, succinate secreted by the helminth *Nippostrongylus brasiliensis*, which is also used in several studies to trigger the tuft cell-induced type 2 immune response,^83,94,95^ is unable to be sensed by tuft cells.^83,94^ It is possible that tuft cells sense helminths through other signaling pathways. As Luo et al. reported,^96^ tuft cells express bitter taste receptors (Tas2rs), which can sense the helminth *Trichinella spiralis* to activate a trimeric G protein to stimulate the phospholipase Cβ2 (Plcβ2)-IP3-Ca^2+^ pathway, which is similar to SUCNR1-SUCNR1-PLC-IP3Ca^2+^ signaling. Hence, not all succinate produced by gut microorganisms can mediate the activation of host mucosal immunity, and the specific underlying causes and mechanisms remain to be elucidated. Nonetheless, succinate potentially serves as a mediator of mutualistic host-protozoan interactions. Furthermore, although it is still controversial whether enteric protozoa (e.g., parabasalids, stramenopiles, and diplomonads) are classified as commensal or pathogens, some findings shed light on the possibility of these pathogens being used to enhance mucosal immunity to resist invading pathogens.^97,98^ Thus, whether some enteric parabasalids are homologous to rodent parabasalid *Tritrichomonas* found in the human intestine, such as *Dientamoeba fragilis*,^98^ and secrete succinate to trigger tuft cell function deserves further exploration.

Thus, whether succinate can be considered a detrimental signal or a factor with a favorable role in host immune regulation, it is clearly related to intestinal homeostasis. When homeostasis is disrupted, succinate may worsen host health. It is not difficult to speculate that regulating succinate concentration is a potential method for regulating host immune function.

## Possible ways to reduce the abnormal accumulation of succinate

**Figure 4.**
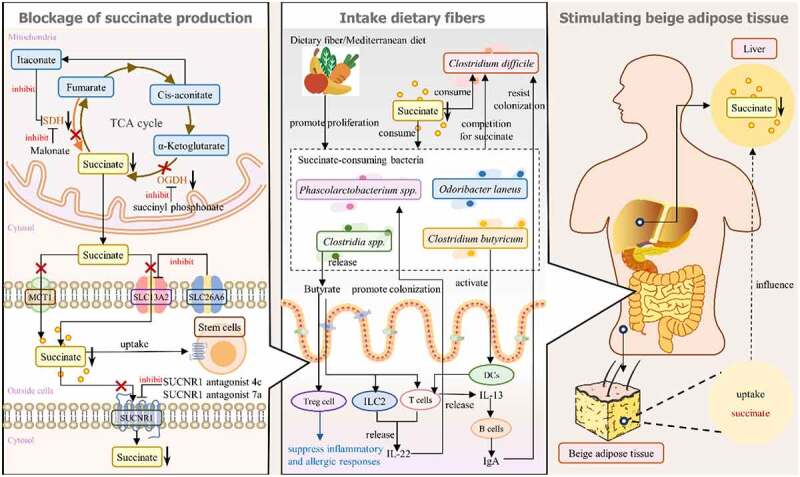
Possible ways to reduce abnormally elevated succinate concentrations. (1) the left section “Blockage of succinate production” shows that methods to block the succinate production pathway in mitochondria include inhibiting SDH activity by promoting itaconate production and inhibiting OGDH activity by using succinyl phosphonate, thereby reducing the production of succinate in the TCA pathway. In addition, blocking the transmembrane transport of succinate can also reduce the level of circulating succinate; that is, increasing SLC26A6 activity to inhibit SLC13A2 or decreasing MCT1 activity may reduce the MCT1-dependent succinate level. Most of the function of succinate is dependent on its binding to SUCNR1 in cells. SUCNR1 antagonist 4c and SUCNR1 antagonist 7a are expected to reduce the negative effects of excessive succinate accumulation by inhibiting SUCNR1 activity. Transplantation of SUCNR1-expressing stem cells can absorb excess succinate. (2) the middle section “Intake dietary fibers” shows that dietary fiber promotes the colonization of succinate-consuming bacteria, which helps to absorb excessive succinate. Some succinate-consuming bacteria can activate T cells, ILC2s and other immune cells to exert immune functions and even promote the proliferation of beneficial succinate-consuming bacteria. Beneficial succinate-consuming bacteria can compete with *Clostridioides difficile*, harmful bacteria that also utilize succinate, and resist their colonization. (3) the right section “Stimulating beige adipose tissue” shows that interaction between tissues is a possible way to reduce the succinate concentration. Beige adipose tissue can absorb succinate in response to cold stimulation, which affects the internal environment of the liver tissue, thereby reducing excessive levels of succinate in the liver. The close connection of the gut–tissue axis suggests that succinate in the intestinal environment can be affected by regulating succinate levels in the internal environment of other tissues.

Based on the above studies, increases in succinate can result from abnormal accumulation of succinate in immune cells caused by an excessive immune response, such as inflammation. Additionally, such increases can be caused by a decline in the number of succinate-consuming gut bacteria. The proinflammatory response induced by an abnormal increase in succinate, in addition to disturbing intestinal homeostasis, also transmits signals to the host and affects the metabolic homeostasis of tissues other than the gut. Abnormal accumulation of succinate can disrupt the enterohepatic circulation of bile acids^34^ and play a core role in gut-lung crosstalk: intestinal I/R causes acute lung injury.^44^ Recently, reducing the succinate concentration has shown promise in treating gut chronic inflammatory diseases^42^ and obesity-related inflammation,^45^ suggesting a new way to alleviate these diseases. Next, we summarize and explore methods to reduce abnormal concentrations of succinate by regulating the production and metabolism of succinate, adjusting the structure of the succinate-related gut microbiota and exploiting the association of succinate in the gut – tissue axis (Figure 4).

### Reduce abnormal accumulation of succinate by affecting succinate synthesis and metabolism pathway

Blocking succinate production by pro-inflammatory immune cells is one way to reduce succinate concentration. In M1 macrophage metabolism, OGDH activity is increased, which promotes the production of succinate from AKG. Inhibiting the expression of OGDH in M1 macrophages by succinyl phosphonate, a specific inhibitor of OGDH, can block succinate production, which prevents the aggravation of aortic aneurysm and dissection (AAD) by high succinate accumulation.^99^ Furthermore, in a partial reversal of purine nucleotide degradation and malate/aspartate interactions in hypoxic microenvironments (e.g., inflammation), where maximal oxygen uptake is reduced to less than 20% of normal levels,^100^ fumarate will act as the terminal electron receptor of the electron transport chain (ETC), which reverses electron flow through SDH. This results in the reduction rate of fumarate exceeding the oxidation rate of succinate and thus leads to succinate accumulation. Malonate is a competitive inhibitor of the carboxylate site of SDHA. It can inhibit succinate accumulation during ischemia and succinate oxidation during reperfusion, thus reducing mitochondrial ROS production and I/R damage.^101^ Itaconate, generated from cis-aconitate by aconitate decarboxylase, can directly inhibit SDH activity in M1 macrophages to block the conversion of fumarate to succinate in a hypoxic microenvironment.^102^ Itaconate plays a complex role similar to that of succinate in the polarization and metabolism of macrophages: it can inhibit both the proinflammatory response of M1 macrophages^103,104^ and the polarization and metabolic reprogramming of M2 macrophages.^105^ Whether there is more association or interaction between itaconate and the change in succinate concentration deserves further study.

Succinate is generally unable to cross the cell membrane except by means of a transporter or by binding to a membrane receptor expressed on the cell to exert its function. Excessive accumulation of succinate is often accompanied by elevated transporter activity.^42^ The Na+-dependent SLC13-family plasma membrane transporters SLC13A2 and SLC13A3, expressed on IECs, colon and kidney cells,^106^ are highly sensitive to succinate.^107^ Of note, the oxalate transporter SLC26A6 binds and inhibits SLC13A2,^108^ and the protein kinase A (PKA) signaling pathway has been confirmed to enhance the expression of SLC26A6,^109^ suggesting a potential ability to regulate extracellular and intracellular succinate homeostasis. However, when the energy demand increases dramatically and the mitochondrial energy supply fails to keep up, the anaerobic system is activated, leading to excessive lactate formation and cellular acidification. This change in pH leads to a change in the chemical structure of succinate, which allows it to cross the cell membrane with the help of monocarboxylate transporter 1 (MCT1).^6,110^ Further studies on methods to inhibit MCT1 activity may provide new ways to reduce MCT1-dependent succinate.

SUCNR1 plays a key role in succinate metabolism. Since the excessive succinate found in a variety of inflammatory conditions exerts effects by reacting with SUCNR1 expressed on immune cells, inhibiting the activity of the SUCNR1 receptor is also a way to reduce the negative effects of excessive succinate on the body. It has been reported that the SUCNR1 antagonist 4c^111^ and the SUCNR1 antagonist 7a^112^ can effectively inhibit SUCNR1 activation. The study of new SUCNR1 antagonists is still ongoing, and whether SUCNR1 antagonists have side effects on the gut microbiota structure needs to be further studied. Further research and development of new SUCNR1 antagonists has broad prospects for the treatment of succinate-related diseases. In addition, transplanting SUCNR1-expressing cells into the inflammatory environment to absorb excess succinate in the internal environment is also a promising method. Mesenchymal stem cell (MSC) transplantation is a promising treatment to restore immune homeostasis by transplantation into injured tissues to repair damaged cells. Multiple studies have found that this approach can reduce succinate concentrations in tissues and create a positive cascade-like effect on other tissues. In brain tissue, when transplanting neural stem cells (NSCs) into the cerebrospinal fluid of autoimmune encephalomyelitis mice, succinate produced by M1 macrophages combines with SUCNR1 on NSCs to upregulate SLC13A3 activity, which is highly sensitive to succinate,^107^ and scavenges succinate in the cerebrospinal fluid, thereby reducing the concentration of succinate. Moreover, succinate prompts NSCs to secrete prostaglandin (PG) E2, which inhibits the proinflammatory effect of M1 macrophages and tends to polarize macrophages into M2 macrophages^113^ to prevent the deterioration of neuroinflammation.^114^ Similarly, transplanted adipose-derived MSCs could also absorb succinate, thereby reducing succinate accumulation and reprogramming macrophages to M2 polarization to alleviate dextran sulfate sodium (DSS)-induced colitis in mice.^115^

### Reduce abnormal accumulation of succinate by regulating gut microbiota composition

Some succinate-consuming bacteria are expected to absorb excess succinate in the environment of intestinal disorders and secrete anti-inflammatory metabolites to regulate host immune function. Succinate favors *Clostridia spp*. colonization, which can protect neonatal mice against diarrhea-causing pathogens, such as *Salmonella typhimurium* and *Citrobacter rodentium*.^31^ In addition, *Clostridia spp*.-producing butyrate promotes the polarization of colonic regulatory T (Treg) cells, which can suppress inflammatory and allergic responses,^116^ and promotes IL-22 release by T cells and ILCs.^117,118^ It has been shown that IL-22 can promote the colonization of *Phascolarctobacterium spp*., succinate-consuming bacteria. Because of its succinate-consuming characteristics, it can compete with *Clostridioides difficile* and minimize dysbiosis symptoms, leading to the reestablishment of a healthy microbiota community, which makes it a promising therapeutic probiotic for *Clostridioides difficile* infections.^119^ In addition, transplantation of *Clostridium butyricum* reduced the abnormal increase in intestinal succinate levels caused by antibiotics, thereby inhibiting *Clostridioides difficile* proliferation and promoting the production of T-cell-dependent pathogen-specific immunoglobulin A (IgA) in the colon, which is involved in the maintenance of host intestinal immune homeostasis.^120^ Dietary intervention, FMT and prebiotic supplementation can improve the gut microbiota composition. Probiotic intervention with *Odoribacter laneus* in obese mice was found to consume circulating succinate.^121^ The Mediterranean diet has been found to increase the ratio of succinate-consuming bacteria (i.e., *Odoribacteraceae* and *Clostridaceae*) to succinate-producing bacteria (i.e., *Prevotellaceae* and *Veillonellaceae*) in obesity and subsequently reduce circulating succinate levels.^45^ The above studies inspire us to transplant specific succinate-consuming bacteria that are beneficial to host health or to administer succinate-consuming probiotics and promote their growth through high-fiber dietary intervention, which is expected to lead to the uptake of excessive succinate and provide new ideas for treating related diseases.

### Regulation of succinate concentrations through the gut – tissue axis

Changes in the composition of gut microbiota can affect the energy metabolism of distal organs and tissues, such as lung, liver, and adipose tissue, through the mucosal immune system, leading to parenteral complications. In addition, these tissues can also influence intestinal homeostasis. During this mutual interaction process, succinate, as a gut microbial metabolite, not only plays essential roles in connecting the gut – tissue axis but also participates in the metabolic processes of tissues. Some tissues can absorb extracellular succinate to reduce succinate concentrations. For example, succinate can be absorbed by brown/beige adipose tissues to increase the expression of the thermogenic gene UCP1, promoting thermogenesis.^122^ Studies on nonalcoholic fatty liver disease (NAFLD) further found that M1 macrophage expression and succinate levels were increased in the livers of mice lacking UCP1 expression, which is also seen in NAFLD. However, when cold stimulation induced the elevation of the content and activity of brown/beige adipose tissues, succinate and SUCNR1 expression in the liver were decreased, alleviating inflammation,^123^ which indicated the involvement of succinate in fat-liver axis metabolism. Since succinate can reach all parts of the host tissues through the blood circulation and the gut – tissue axis is interconnected and the components affect each other, we speculate that the excessive succinate content in the intestinal environment can be reduced by targeting other tissues to absorb succinate for tissue metabolism to alleviate intestinal diseases. However, due to the complexity and unpredictability of the interaction between the gut and other tissues, it is very challenging to regulate the internal environment of one tissue to affect another tissue. Nonetheless, with increasing research on the gut – tissue axis, it is believed that in the near future, targeted regulation of succinate metabolism will become an effective method for the treatment of intestinal diseases and parenteral complications.

In short, from the perspective of the production and metabolism pathway of succinate, the regulation of gut microbiota structure, and the relationship between the gut and other tissues, we have considered possible methods to reduce the excessive accumulation of succinate in the context of immune disorders. Although these are conjectures based on partial research, combined with current scientific and technological advances, there is the possibility of in-depth study. The association between abnormally increased succinate and various types of inflammation has been a focus. In particular, the intestinal succinate level and SUCNR1 expression in IBD patients are higher than those in healthy individuals.^9,10^ Whether the relationship between intestine-produced succinate and IBD makes it possible to apply succinate in the prediction and treatment of IBD deserves further study. In recent years, it has been reported that creeping mesenteric fat is a special adipose tissue that attempts to block leakage sites in intestinal lesions and prevent intestinal bacteria from entering the blood. Gut microbiota-produced succinate was found to be involved in the browning process of creeping mesenteric fat, thereby reversing fibrosis caused by local chronic inflammation and slowing the deterioration of CD.^124^ This opens up the possibility of applying succinate as a therapeutic strategy in IBD.

## Conclusion and future prospects

Succinate, as a product of host metabolism, can control the growth, differentiation, and function of immune cells due to the wide expression of the succinate receptor SUCNR1. Certain members of the intestinal microbiota provide succinate for their host to activate immune function and maintain body health via the gut – tissue axis. Moreover, the association with pathogens further reflects the key role of succinate in maintaining intestinal homeostasis. Many previous studies on succinate have revealed that abnormal increases in succinate increase the risk of immune disorders linked to diseases such as inflammation, tumors, and cancer. However, several recent studies have shown that lowering succinate concentrations appears to alleviate or treat inflammatory diseases. Although the feasibility of decreasing succinate concentration is confirmed by these studies in vitro, doing so in vivo is more complex. Intestinal homeostasis is related to host immune cells and affected by the community structure of the intestinal microbiota, which makes the pathogenesis and treatment of gut diseases complicated and difficult. For example, although reducing excessive succinate levels from the perspective of succinate production and metabolic pathways have the potential to alleviate inflammation, it may have side effects on the dynamic structure of the gut microbiota. Human intervention is bound to break the original intestinal microbial structure, and through a series of chain reactions, the intestinal microbial structure will ultimately reach an equilibrium state again. Whether these changes will cause other negative effects is a difficult question that needs further research. Therefore, we hypothesized that the screening and development of specific succinate-consuming probiotics or transplantation of specific beneficial succinate-consuming bacteria through FMT and promoting their growth through dietary strategies would be relatively stable and safe succinate-lowering therapies.

## Data Availability

The data that support the findings of this study are available from the corresponding author, [CQG], upon reasonable request. https://orcid.org/0000–0001–9119–1233.

## Abbreviations

AAD: aortic aneurysm and dissection
AKG: α-ketoglutarate
APCs: antigen-presenting cells
ASCT: acetate:succinate CoA-transferase
BMDMs: bone-marrow-derived macrophages
CD: Crohn’s disease
CO_2_: carbon dioxide
CoQ: coenzyme Q
DCs: dendritic cells
DSS: dextran sulfate sodium
ERK1/2: extracellular signal-regulated kinases 1 and 2
ETC: electron transport chain
FBPase: fructose-1,6-bisphosphatase
FMT: fecal microbiota transplantation
GABA: γ-aminobutyric acid
HIF-1α: hypoxia-inducible factor-1α
I/R: ischemia/reperfusion
IBD: inflammatory bowel disease
IDH: isocitrate dehydrogenase
IECs: intestinal epithelial cells
IgA: immunoglobulin A
ILC2s: type 2 innate lymphoid cells
iMoDCs: immature monocyte-derived DCs
IP3: inositol trisphosphate
MCM: methylmalonyl-CoA mutase
MCT1: monocarboxylate transporter 1
MMA: methylmalonate
MSC: mesenchymal stem cell
NAFLD: nonalcoholic fatty liver disease.
NSCs: neural stem cells
OAA: oxaloacetate
OGDH: 2-oxoglutarate dehydrogenase
OXPHOS: oxidative phosphorylation
PEP: phosphoenolpyruvate
PEPCK: phosphoenolpyruvate carboxykinase
PGE2: prostaglandin E2
PHD: proline hydroxylase
PKA: protein kinase A
PLC: phospholipase C
Plcβ2: phospholipase Cβ2
RA: rheumatoid arthritis
RELMβ: resistin-like protein β
ROS: reactive oxygen species
SCFAs: short-chain fatty acid
SCS: succinyl-CoA synthetase
SDH: succinate dehydrogenase
SUCNR1: succinate receptor 1
Tas2rs: tuft cells express bitter taste receptors
TCA: tricarboxylic acid cycle
Th cells: T helper cells
TLRs: Toll-like receptors
Treg cells: regulatory T cells
TRPM5: transient receptor potential cation channel subfamily M member 5
UQ: ubiquinone
UQH_2_: ubiquinol
